# The critical role of lipopolysaccharide in the upregulation of aquaporin 4 in glial cells treated with Shiga toxin

**DOI:** 10.1186/s12929-015-0184-5

**Published:** 2015-09-18

**Authors:** Naotoshi Sugimoto, Hue Leu, Natsumi Inoue, Masaki Shimizu, Tomoko Toma, Mondo Kuroda, Takekatsu Saito, Taizo Wada, Akihiro Yachie

**Affiliations:** Department of Physiology, Graduate School of Medical Science, Kanazawa University, 13-1 Takara-machi, Kanazawa, 920-8640 Japan; Department of Pediatrics, Graduate School of Medical Science, Kanazawa University, Kanazawa, Japan; Dan Phuong General Hospital, Hanoi, Vietnam

**Keywords:** Encephalopathy, Aquaporin 4 (AQP4), Lipopolysaccharide (LPS), Shiga toxin (Stx), Nuclear factor-κB (NF-κB) signaling

## Abstract

**Background:**

In 2011, there was an outbreak of Shiga toxin-producing *Escherichia coli* (STEC) infections in Japan. Approximately 62 % of patients with hemolytic-uremic syndrome also showed symptoms of encephalopathy. To determine the mechanisms of onset for encephalopathy during STEC infections, we conducted an *in vitro* study with glial cell lines and primary glial cells.

**Results:**

Shiga toxin 2 (Stx-2) in combination with lipopolysaccharide (LPS), or LPS alone activates nuclear factor-κB (NF-κB) signaling in glial cells. Similarly, Stx-2 in combination with LPS, or LPS alone increases expression levels of aquaporin 4 (AQP4) in glial cells. It is possible that overexpression of AQP4 results in a rapid and increased influx of osmotic water across the plasma membrane into cells, thereby inducing cell swelling and cerebral edema.

**Conclusions:**

We have showed that a combination of Stx-2 and LPS induced apoptosis of glial cells recently. Glial cells are indispensable for cerebral homeostasis; therefore, their dysfunction and death impairs cerebral homeostasis and results in encephalopathy. We postulate that the onset of encephalopathy in STEC infections occurs when Stx-2 attacks vascular endothelial cells of the blood–brain barrier, inducing their death. Stx-2 and LPS then attack the exposed glial cells that are no longer in contact with the endothelial cells. AQP4 is overexpressed in glial cells, resulting in their swelling and adversely affecting cerebral homeostasis. Once cerebral homeostasis is affected in such a way, encephalopathy is the likely result in STEC patients.

**Electronic supplementary material:**

The online version of this article (doi:10.1186/s12929-015-0184-5) contains supplementary material, which is available to authorized users.

## Background

An outbreak of the Shiga toxin (Stx)-producing *Escherichia coli* (STEC) infections occurred in the Hokuriku District of Japan in 2011 [1–4]. Around 62 % of hemolytic-uremic syndrome (HUS) patients showed symptoms of encephalopathy [1, 2]. Unfortunately, five of these patients passed away [1–3]. It was previously reported that the incidence of encephalopathy for HUS patients is less than 50 % [5–7]; therefore, 62 % could be considered a high incidence rate [1, 2]. Encephalopathy does not refer to a single disease, but is a syndrome of brain dysfunction with organic and inorganic causes, including cytokine storm, toxic reaction and neurotransmitter effects [8, 9]. Cerebral edema is often observed as a part of encephalopathy during STEC infections [1–7, 10].

Glial cells are important cells that maintain cerebral homeostasis, and are functional components of the blood–brain barrier (BBB) [11]. Glial cells also regulate water metabolism, via aquaporin 4 (AQP4), in the encephalon [12, 13]. Therefore, glial cell hypofunction and death impairs cerebral homeostasis, and is thought to result in encephalopathy [11]. Stx is known to adversely affect vascular endothelial cells, which are components of the BBB, and induce their death [14]; however, the effects of Stx on glial cells are unclear.

We recently reported that Stx decreases the ability of glial cells to tolerate heat, and that they die when exposed to a combination of Stx and heat shock *in vitro* [15]. During the 2011 STEC outbreak in Japan, three of the four inpatients with encephalopathy at Tonami City Hospital exhibited a high fever [4]. STEC are gram-negative bacilli, and contain lipopolysaccharide (LPS) as a component of their cell walls [16]. LPS is an exogenous pyrogen that induces fever and is often referred to as an endotoxin [17]. It is thought that LPS might play a significant role in STEC infection-induced encephalopathy. To determine the mechanisms of onset for encephalopathy during STEC infections, we conducted various *in vitro* experiments with glial cell lines and primary glial cells. We also investigated the effects of Stx and LPS on glial cells *in vitro*.

## Methods

### Chemicals

Dulbecco’s modified Eagle’s medium (DMEM) and lipopolysaccharide (LPS) were obtained from Wako Pure Chemical Industries, Ltd (Osaka, Japan). Shiga toxin-2 was obtained from Nacalai Tesque (Kyoto, Japan). Fetal bovine serum (FBS) and interleukin-1 receptor-associated kinase (IRAK)-1/4 inhibitor were obtained from Invitrogen Corporation (Carlsbad, CA, USA) and Calbiochem (Billerica, MA, USA), respectively. Anti-phospho-specific nuclear factor-κB (NF-κB) p65 (Ser536), anti-NF-κB p65, anti-phospho-specific extracellular signal-regulated kinase (ERK) (Thr202/Tyr204), anti-ERK, anti-β-actin, horseradish peroxidase-conjugated anti-rabbit IgG, and anti-mouse IgG were purchased from Cell Signaling Technology (Danvers, MA, USA). Anti-COX2 antibody was obtained from Cayman Chemical Company (Ann Arbor, MI, USA). An antibody against AQP4 was purchased from Millipore (Billerica, MA, USA).

### Cell culture

B92 rat glial cells and primary rat glial cells [18] were provided by Dr. Ohno-Shosaku (Kanazawa University, Japan) [19]. The glial fibrillary acidic protein (GFAP), a marker of astrocyte, expression was detected in primary cells that Dr. Ohno-Shosaku provided, indicating astrocytes (Additional file 1: Figure S1). Cells were maintained in DMEM containing 10 % FBS at 37 °C/5 % CO_2_.

### Western blotting analysis

We performed western blotting as previously described [20]. Band density analysis was conducted using densitometry scanning with ImageJ (http://imagej.nih.gov/ij/).

### Reverse transcription polymerase chain reaction (RT-PCR) assays

To evaluate the mRNA expression patterns of cyclooxygenase 1 (COX1) and COX2 in cells, we used RT-PCR assays. Briefly, RNA was extracted from cells and reverse transcribed using the reverse transcriptase ReverTra Ace® (Toyobo, Tokyo, Japan). Samples were then subjected to PCR using LA Taq DNA Polymerase (Takara, Tokyo, Japan) with primers specific for COX1 (5′-AAT GCC ACC TTC ATC CGA GA-3′ and 5′-TGG GTG AAG TGT TGT GCA AAG-3′), COX2 (5′-CAG CAA ATC CTT GCT GTT CC-3′ and 5′-GTG AAG TGC TGG GCA AAG AAT-3′), and β-actin (5′-ATG GTG GGT ATG GGT CAG AAG-3′ and 5′-CTG GGG TGT TGA AGG TCT CAA-3′).

### Statistical analysis

Data are presented as the mean ± SEM from at least three independent experiments. Statistical analysis was conducted using analysis of variance (ANOVA) followed by a post-hoc Dunnett’s test. Results were considered statistically significant when *p*-values were less than 0.05 or 0.01.

## Results

### Stx-2 with LPS and LPS alone, but not Stx-2 alone, phosphorylates NF-κB in B92 glial cells

**Fig. 1:**
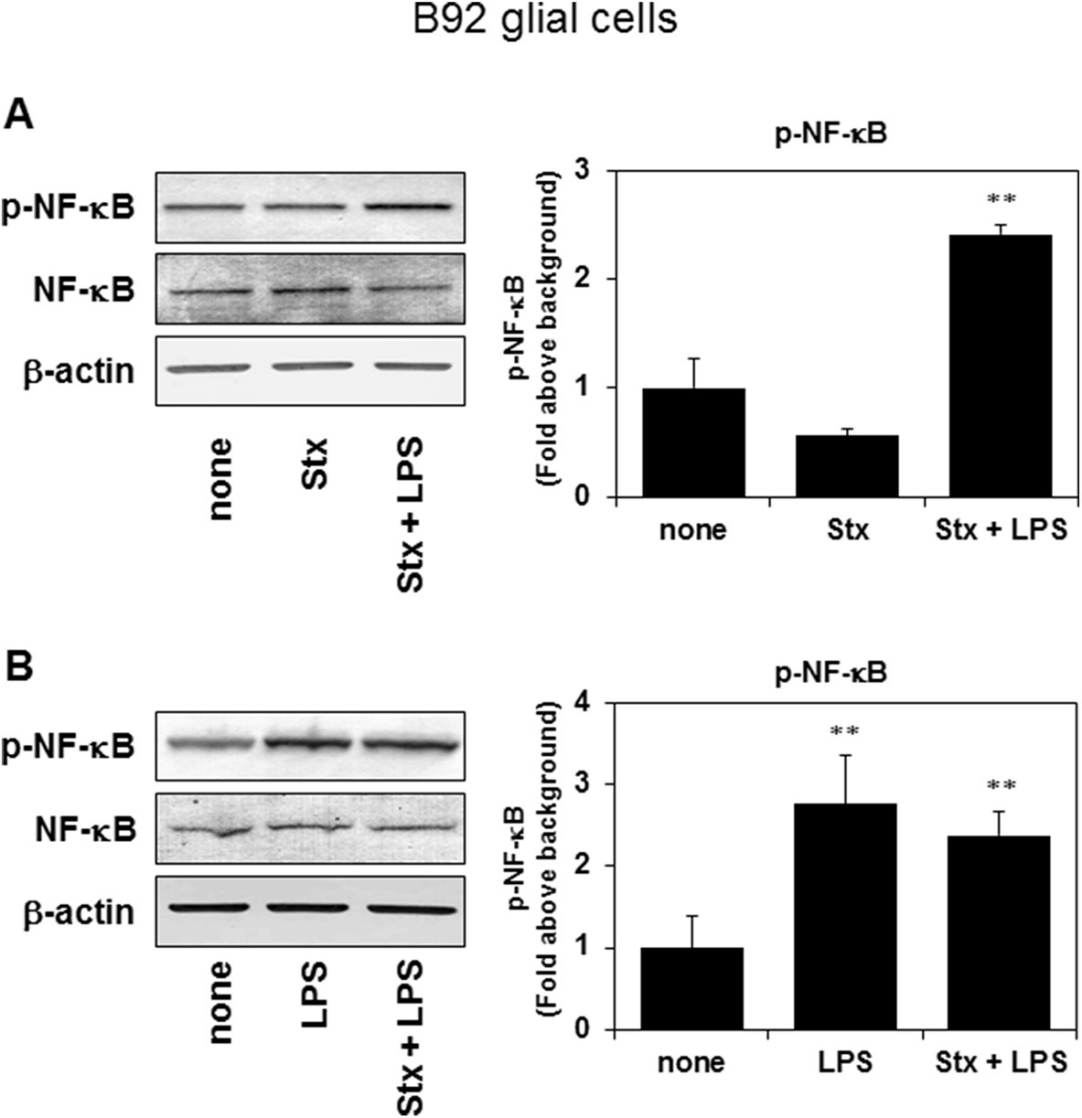
Phosphorylation of NF-κB was increased in B92 glial cells, 20 min after stimulation with Stx-2 (3 ng/mL) and LPS (0.1 μg/mL), but not Stx-2 alone (3 ng/mL) (**a**). Phosphorylation of NF-κB was increased in B92 glial cells, 20 min after stimulation with Stx-2 and LPS, or LPS alone (0.1 μg/mL) (**b**). Values are presented as means ± SEM. **P < 0.01

We first examined the effects of Stx-2/LPS alone or in combination with LPS on NF-κB signaling in cultured glial cells. The concentration of Stx-2 and LPS were decided by previous our results [15]. Stx-2 (3.0 ng/mL) on its own failed to phosphorylate NF-κB (Fig. 1a). However, Stx-2 and LPS combined resulted in the phosphorylation of NF-κB (Fig. 1a, b). Also, LPS alone stimulates the phosphorylation of NF-κB (Fig. 1b).

### Stx-2 with LPS and LPS alone, but not Stx-2 alone, upregulates COX2 expression in B92 glial cells

**Fig. 2:**
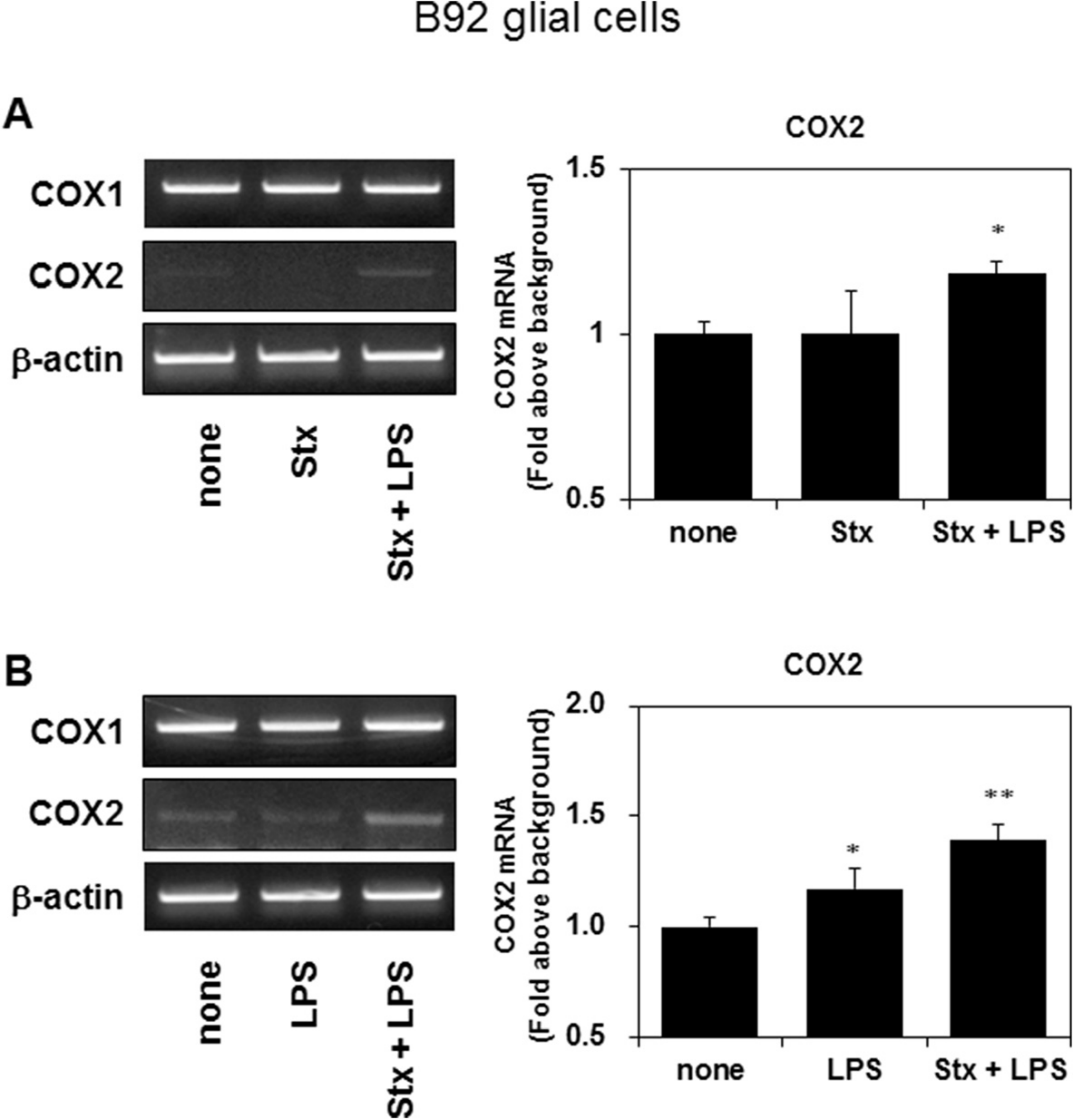
COX2 mRNA expression was increased in B92 glial cells, 24 h after stimulation with Stx-2 and LPS, but not Stx-2 alone (**a**). COX2 mRNA expression levels were increased in B92 glial cells, 24 h after stimulation with Stx-2 and LPS, or LPS alone (**b**). Values are presented as means ± SEM. *P < 0.05, **P < 0.01

NF-κB signaling plays an important role in the inflammatory response, including the regulation of COX2 expression [21]. Stx-2 failed to increase COX2 expression, but when combined with LPS, the expression of COX2 was upregulated in B92 glial cells (Fig. 2a, b). Also, LPS alone stimulates the expression of COX2 (Fig. 2b). Stx-2 or LPS alone, or Stx-2 combined with LPS did not affect COX1 expression in B92 glial cells (Fig. 2a, b).

### Stx-2 alone or in combination with LPS, but not LPS alone, inhibits the phosphorylation of ERK in B92 glial cells

**Fig. 3:**
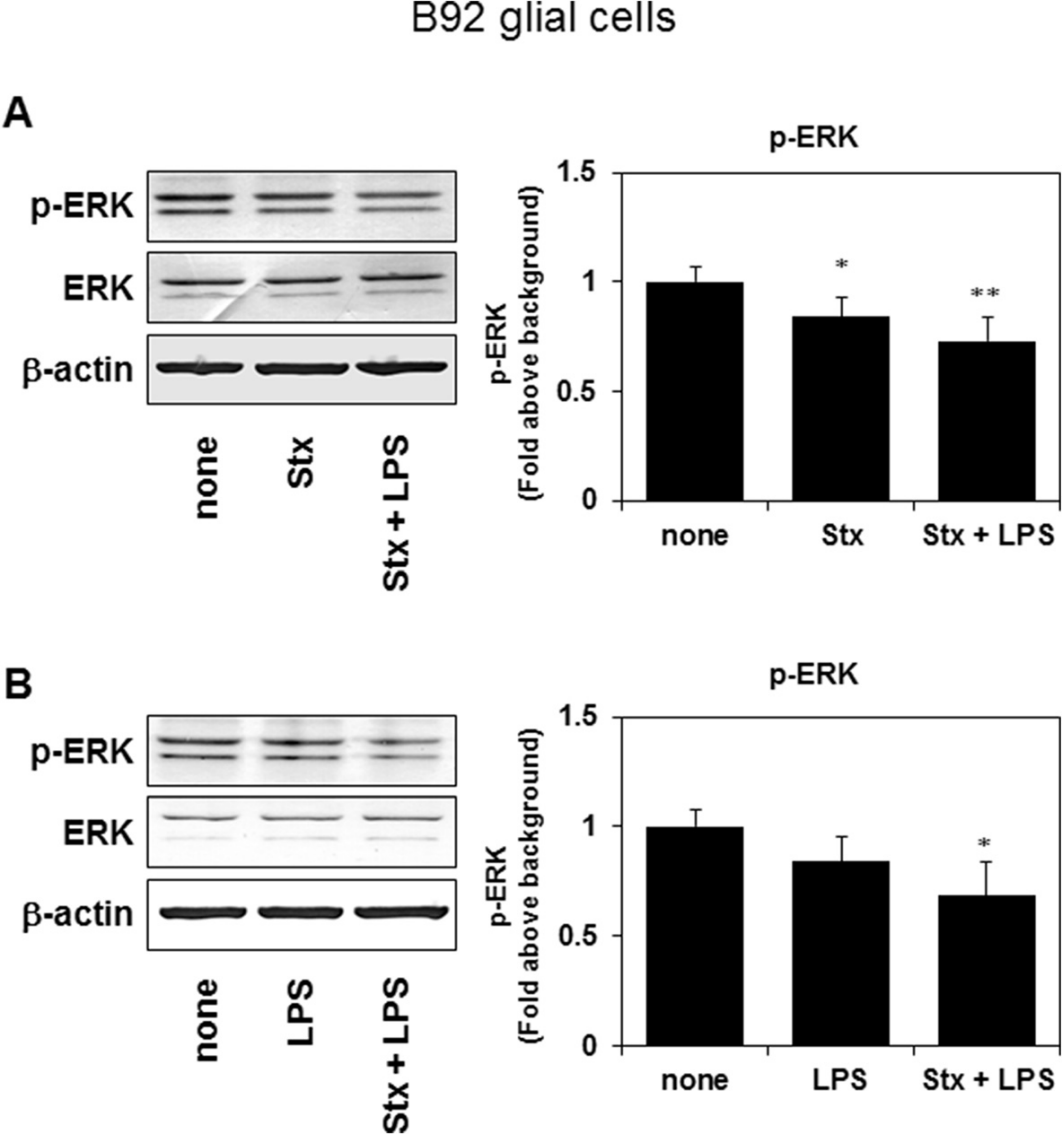
Phosphorylation of ERK was decreased in B92 glial cells, 20 min after stimulation with Stx-2 and LPS, or Stx-2 alone (**a**). Phosphorylation of ERK was decreased in B92 glial cells, 20 min after stimulation with Stx-2 and LPS, but not LPS alone (**b**). Values are presented as means ± SEM. *P < 0.05, **P < 0.01

We examined the effects of Stx-2 and/or LPS on ERK activity. The ERK signaling pathway is crucial for the regulation of cell growth [22, 23]. Stx-2 in combination with LPS decreased the levels of phosphorylated ERK to a greater extent than Stx-2 alone (Fig. 3a, b). However, LPS on its own failed to inhibit phosphorylation of ERK (Fig. 3b).

### Stx-2 with LPS and LPS alone upregulates phosphorylation of NF-κB, and the expression of COX2 and AQP4 in primary glial cells

**Fig. 4:**
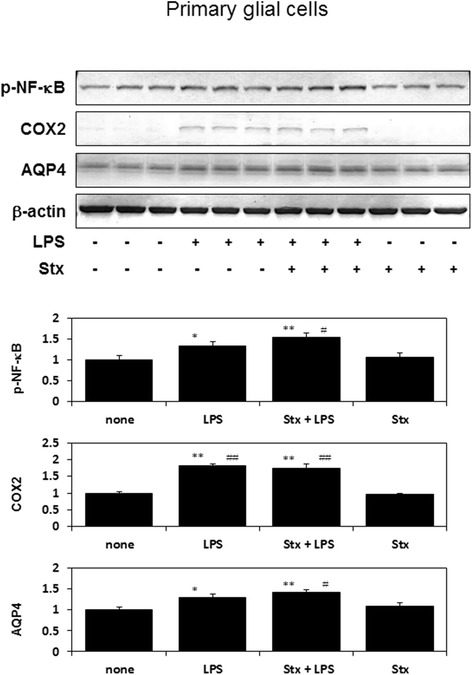
Phosphorylation of NF-κB, COX2 and AQP4 protein levels were increased in primary glial cells, 36 h after stimulation with Stx-2 and LPS, or LPS alone. Values are presented as means ± SEM. *P < 0.05, **P < 0.01 vs. none control, ^#^ *P* < 0.05, ^##^ *P* < 0.01 vs. Stx treatment

Stx-2 in combination with LPS and LPS alone increased phosphorylation levels of NF-κB, and expression levels of COX2; however, Stx-2 alone did not affect those levels of these proteins (Fig. 4). While, AQP4 is expressed on glial cells and participates in the transportation of water through the cell membrane. Furthermore, AQP4 is also related to the incidence of cerebral edema. We examined the effects of Stx-2 and/or LPS on AQP4 in primary glial cells. The combination of Stx-2 and LPS and LPS alone increased the expression of AQP4, while Stx-2 alone did not affect AQP4 expression in primary glial cells (Fig. 4). Our results indicate that LPS plays a critical role in the upregulation of AQP4 in glial cells.

### IRAK-1/4 inhibitor attenuates LPS-induced AQP4 expression in primary glial cells

**Fig. 5:**
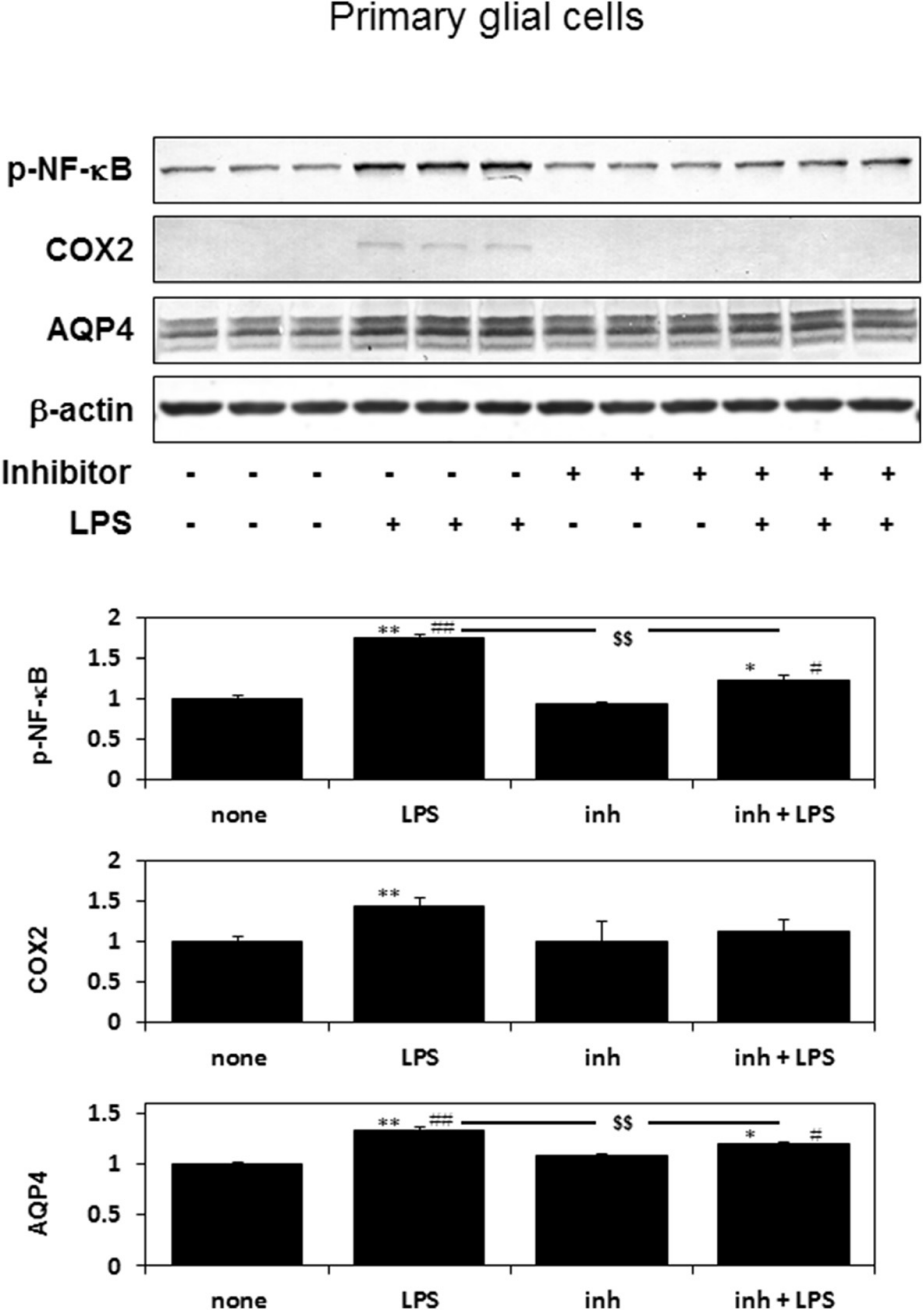
IRAK-1/4 inhibitor attenuates LPS-induced phosphorylation of NF-κB, COX2 and AQP4 protein expressions in primary glial cells, 36 h after stimulation with LPS following IRAK-1/4 inhibitor (1 μM) pretreatment. Values are presented as means ± SEM. ^*^ *P* < 0.05, ^**^ *P* < 0.01 vs. none control, ^#^ *P* < 0.05, ^##^ *P* < 0.01 vs. inhibitor alone, ^$$^ *P* < 0.01 vs. LPS treatment

Next, we demonstrate that whether NF-κB signaling plays an important role in the AQP4 expression in primary glial cells. IRAK-1/4 is an upstream molecule of NF-κB [24]. IRAK-1/4 inhibitor attenuates LPS-induced phosphorylation of NF-κB, COX2 and AQP4 expressions in primary glial cells (Fig. 5).

## Discussion and conclusions

Encephalopathy is recognized as a major determinant of mortality during the acute phase of STEC infections. Although the pathogenesis of CNS complications is unclear, disruption of the BBB and neuronal disturbances are observed during the acute phase of STEC infections [1–7, 10]. Glial cells are the most numerous cells in the brain, and are major cellular components of the mature BBB. Furthermore, glial cells are indispensable for cerebral homeostasis [11]; therefore, glial cells likely play a critical role in influencing encephalopathy during STEC infections. We found that treatment of glial cells with Stx-2 in combination with LPS and LPS alone resulted in an increase in AQP4 expression in NF-κB dependent manner.

Aquaporins (AQPs) are a family of integral membrane proteins, closely associated with water transport, with at least 13 members identified in mammals [25, 26]. AQPs play an important role in water permeation [27], with their expression inducing 10- to 100-fold increases in the water permeability of cells [28]. AQP4 is expressed on glial cells and maintains the homeostasis of the cerebral environment by regulating of water permeation [12]. However, overexpression of AQP4 can result in a rapid influx of osmotic water across the plasma membrane into glial cells, resulting in cell swelling and cerebral edema [29–31]. It is worth noting that cerebral edema due to infection does not occur in mice where AQP4 has been knocked out [32].

We showed that neither LPS nor Stx results in the death of glial cells [15]. However, when Stx and LPS are combined, the death of glial cells is induced [15]. Expression of AQP4 occurs during NF-κB signal transduction, similar to COX2 expression [33–35]. In the current study, Stx on its own did not activate NF-κB (Figs. 1 and 4), and failed to induce AQP4 expression (Fig. 4). However, Stx in combination with LPS or LPS alone activated NF-κB (Figs. 1 and 4) and induced AQP4 expression (Figs. 4 and 5). IRAK-1/4 inhibitor attenuates LPS-induced AQP4 expression (Fig. 5), indicating AQP4 transcription acts downstream of NF-κB signal transduction. STEC stimulates the innate immune system of a host, thereby leading to inflammatory responses. LPS is the main cell wall component of STEC; therefore LPS and Stx are important factors during STEC infections. LPS stimulates TLR4, and activates a number of inflammatory pathways, including activation of NF-κB [36]. NF-κB is a key transcription factor involved in inflammatory responses, including the induction of COX2, and also plays a critical role in cell growth [23]. From our findings, we have shown that LPS plays a significant role in modifying of virulence of Stx.

**Fig. 6:**
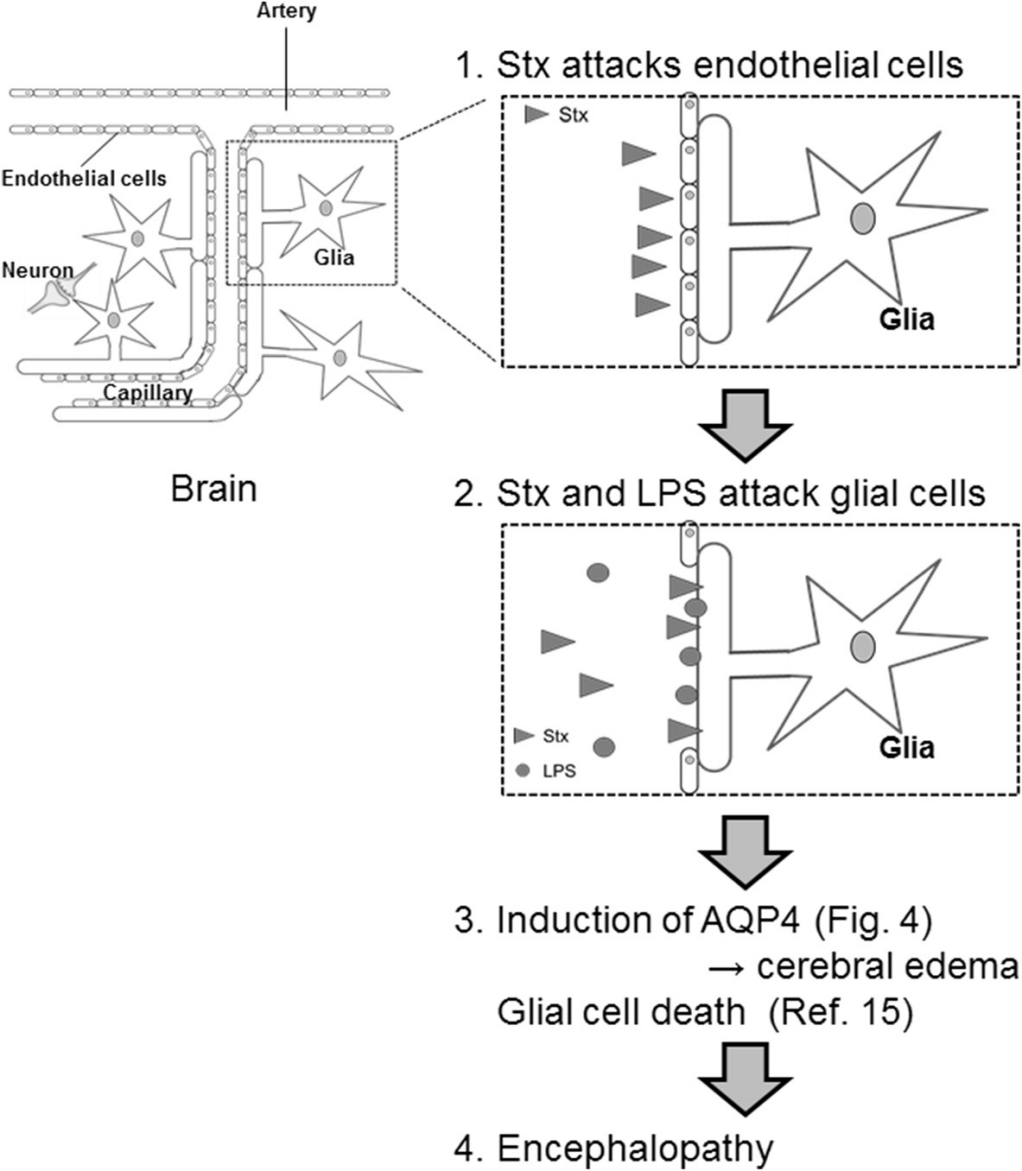
Schematic outlining how Stx and LPS induce encephalopathy during STEC infections

Many patients with encephalopathy were reported during the 2011 Japanese outbreak of STEC infections [1–7, 10]. Although the mechanism(s) of the onset of encephalopathy during these STEC infections were unclear at the time, we believe we have elucidated at least one mechanisms in the current study (Fig. 6). When Stx and LPS are present in the blood, Stx attacks vascular endothelial cells that form the BBB, and induce their death. In turn, Stx and LPS attack the exposed glial cells that are no longer in contact with endothelial cells. Following on from this, AQP4 expression is upregulated in glial cells, which results in their death. When AQP4 is overexpressed, it is thought to cause a cerebral edema, and glial cell death likely adversely affects the cerebral homeostasis. The overall result of these events is encephalopathy in STEC patients.

## Additional file

Additional file 1: Figure S1. GFAP was expressed in primary glial cells. (BMP 1147 kb)

## Abbreviations

AQP4: Aquaporin 4
BBB: Blood–brain barrier
COX: Cyclooxygenase
ERK: Extracellular signal-regulated kinase
GFAP: Glial fibrillary acidic protein
HUS: Hemolytic-uremic syndrome
IRAK: Interleukin-1 receptor-associated kinase
LPS: Lipopolysaccharide
STEC: Shiga toxin-producing *Escherichia coli*
Stx: Shiga toxin
TLR4: Toll-like receptor 4
