# Supplementary figures and images for: The critical role of lipopolysaccharide in the upregulation of aquaporin 4 in glial cells treated with Shiga toxin
Source: J Biomed Sci. 2015 Sep 18;22(1):78. doi: 10.1186/s12929-015-0184-5 (PMC4575422; doi:10.1186/s12929-015-0184-5)

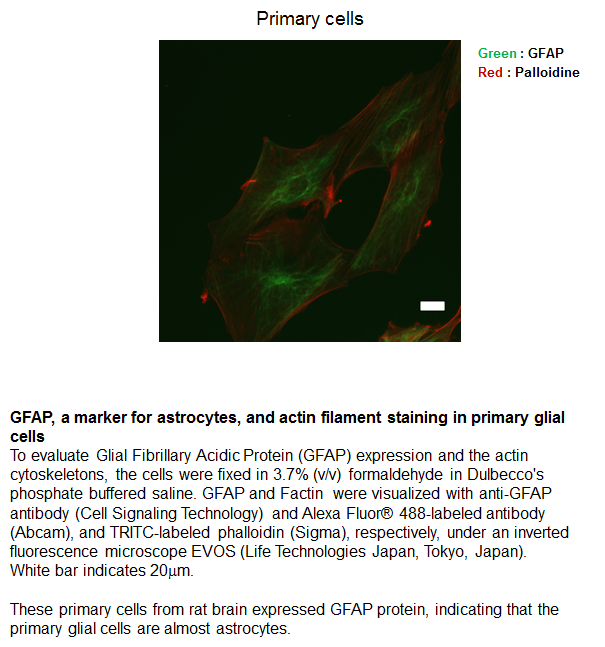

Supplement: Additional file 1: Figure S1. — GFAP was expressed in primary glial cells. (BMP 1147 kb) [file 12929_2015_184_MOESM1_ESM.bmp]
